# Supplementary material for: Probabilities of Multilocus Genotypes in SIB Recombinant Inbred Lines
Source: Front Genet. 2019 Oct 1;10:833. doi: 10.3389/fgene.2019.00833 (PMC6781035; doi:10.3389/fgene.2019.00833)
Supplement: Supplementary file 1 [file DataSheet_1.pdf]

Supplementary Material

1 SUPPLEMENTARY MATHEMATICS

Here we prove the equation

$$N_Q(L) = 2^{L-2}(2^{L-1} + 1) \tag{S1}$$

Proof: If L is the number of loci, there are 4^L IBD (identical by descent) probabilities $Q(i_1, i_2, \ldots i_L)$ where $i_l = 0, 1, 2$ or 3 and furthermore these probabilities add up to 1. A number of these probabilities are equal because of two symmetries: (1) the two homologous chromosomes in each individual play identical roles, and (2) the siblings play identical roles (assuming no sex-dependence of meiosis, so that the recombination rates $r_{l,l'}$ are sex-independent). It is thus appropriate to use only one representative of each equivalence class generated by these symmetries. A way to do this is to first impose that this representative have its first index, i_1, equal to zero. Second, we can then specify exactly one element in each class by imposing that the indices of the representative Q's have either

1. $i_l \in \{0, 1\}\, \forall l \in \{2, .., L\}$, *or*
2. $i_l \in \{0, 1\}\, \forall l \in \{2, .., K-1\}$, $i_K = 2$ and $i_l \in \{0, 1, 2, 3\}\, \forall l \in \{K+1, .., L\}$

The number of equivalence classes and thus of Q's to consider is then

$$N_Q(L) = 2^{L-1} + \sum_{l=2}^{L} 2^{l-2} 4^{L-l} = 2^{L-1} + 2^{2L-2} \sum_{l=2}^{L} 2^{-l} \tag{S2}$$

Given that $\sum_{l=2}^{L} 2^{-l}$ is a geometric progression of common ratio 2^{-1} from 2 to L, the sum of its terms can be expressed as:

$$\sum_{l=2}^{L} 2^{-l} = \frac{2^{-2} - 2^{-(L-1)}}{1 - 2^{-1}} = 2^{-1} - 2^{-L} \tag{S3}$$

Substituting S3 in S2, we get

$$N_Q(L) = 2^{L-1} + 2^{2L-2}(2^{-1} - 2^{-L}) = 2^{L-1} + 2^{2L-3} - 2^{L-2} \tag{S4}$$

Factorizing with respect to 2^{L-2} and after simplification, this gives

$$N_Q(L) = 2^{L-2}(1 + 2^{L-1}). \tag{S5}$$

2 IMPUTING USING THE EXACT RIL PROBABILITIES

We compared the performance of the missForest R package to our new approach that exploits the exact multilocus genotype probabilities. As mentioned in the Main, our method is based on focusing on missing data forming blocks of consecutive markers. When the block is large (this happens stochastically), it may be impossible in practice (for time and memory) to compute the needed multilocus genotype probabilities. To overcome this difficulty, we have implemented a “divide and conquer” method whereby inside the block we first focus on a subset of just 3 of those markers. After imputation is done on these 3, imputation requiring calculating multilocus probabilities involving 5 loci because of the flanking markers, we proceed to consider the remaining markers with missing data; these are now organized into one or more blocks of smaller size. The divide and conquer process can thus be repeated iteratively until there are no more markers to impute. A choice has to be made in the “divide” step for selecting the 3 most relevant markers. We do that by a bottom-up greedy approach where markers are successively removed, one step at a time. At each step, we first find the 2 markers that are closest (in this test we include the flanking markers and distances are in cM); if only one marker has missing data, we remove it; if both have missing data, we remove the one which is closest to its other adjacent marker.

For each value of the fraction of missing data (0.1, 0.2, 0.3, 0.4, 0.5 and 0.7), and for each replicate of a SIB RIL population (cf. the scatter plot of the Main), we determined the fraction of missing data that were incorrectly imputed in each method. Based on these replicates, Fig. S1 provides the box plots for each level of missing data studied. Clearly, the distributions of values hardly overlap, allowing us to conclude that using the exact multilocus RIL probabilities leads to a big improvement.

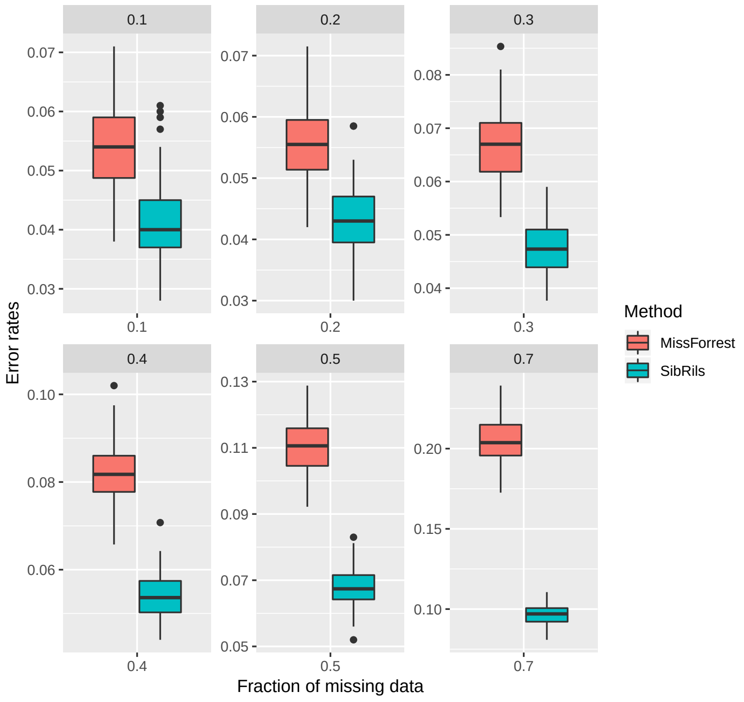

Figure S1: Box plots to compare imputation error rates between the missForest machine learning algorithm and our approach using the *exact* values of the multilocus genotype probabilities. The fraction of missing data applied to the datasets are given at the top of each plot. For almost all cases, there is hardly any overlap between the distributions of the two algorithms, the exact approach is systematically better.

3 THE SELF-CONSISTENT EQUATIONS FOR THREE LOCI

Here we provide the coefficients entering each of the $N_Q(L) = 10$ self-consistent equations for $L = 3$.

3.1 The self consistent equation for $Q(0,0,0)$

Figure S2 displays the 8 factors in the self-consistent equation for $Q(0,0,0)$:

$$Q(0,0,0) = \frac{1}{2}(1-r_{12})(1-r_{23})[Q(0,0,0)+Q(2,2,2)] + \frac{1}{4}(1-r_{12})[Q(0,0,2)+Q(2,2,0)] + \frac{1}{4}(1-r_{13})[Q(0,2,0)+Q(2,0,2)] + \frac{1}{4}(1-r_{23})[Q(0,2,2)+Q(2,0,0)] \quad \text{(S6)}$$

After use of symmetry to keep only non-equivalent Qs, this leads to

$$Q(0,0,0) = (1-r_{12})(1-r_{23})Q(0,0,0) + \frac{1}{2}(1-r_{12})Q(0,0,2) + \frac{1}{2}(1-r_{13})Q(0,2,0) + \frac{1}{2}(1-r_{23})Q(0,2,2)$$

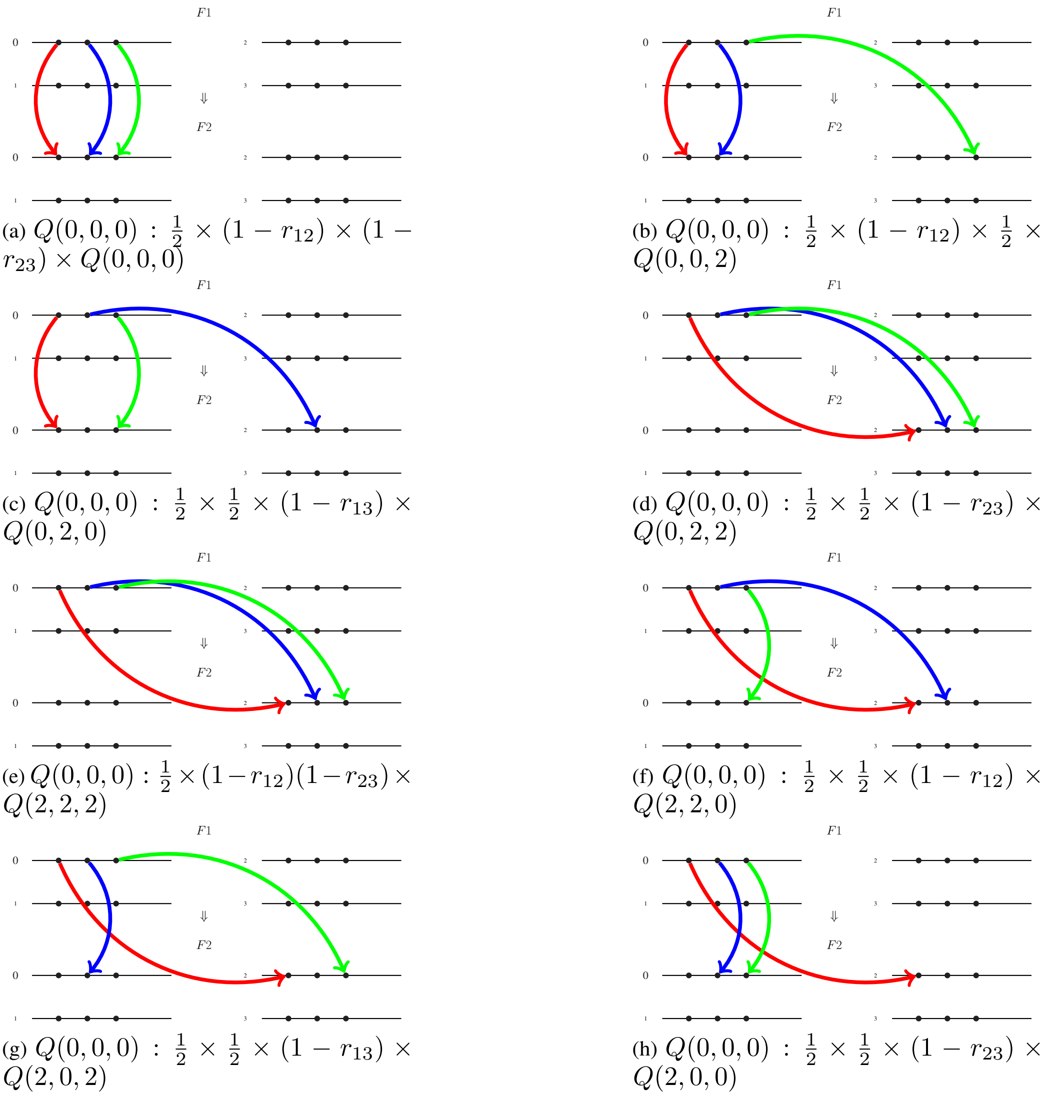

(a) $Q(0,0,0)$: $\frac{1}{2} \times (1-r_{12}) \times (1-r_{23}) \times Q(0,0,0)$

(b) $Q(0,0,0)$: $\frac{1}{2} \times (1-r_{12}) \times \frac{1}{2} \times Q(0,0,2)$

(c) $Q(0,0,0)$: $\frac{1}{2} \times \frac{1}{2} \times (1-r_{13}) \times Q(0,2,0)$

(d) $Q(0,0,0)$: $\frac{1}{2} \times \frac{1}{2} \times (1-r_{23}) \times Q(0,2,2)$

(e) $Q(0,0,0)$: $\frac{1}{2} \times (1-r_{12})(1-r_{23}) \times Q(2,2,2)$

(f) $Q(0,0,0)$: $\frac{1}{2} \times \frac{1}{2} \times (1-r_{12}) \times Q(2,2,0)$

(g) $Q(0,0,0)$: $\frac{1}{2} \times \frac{1}{2} \times (1-r_{13}) \times Q(2,0,2)$

(h) $Q(0,0,0)$: $\frac{1}{2} \times \frac{1}{2} \times (1-r_{23}) \times Q(2,0,0)$

Figure S2: The graphical representation of the factors multiplying each Q on the right-hand side of Eq. S6 for $Q(0,0,0)$.

3.2 The self consistent equation for $Q(0,0,1)$

Figure S3 displays the 8 factors in the self-consistent equation for $Q(0,0,1)$:

$$Q(0,0,1) = \frac{1}{2}(1-r_{12})r_{23}[Q(0,0,0)+Q(2,2,2)] + \frac{1}{4}(1-r_{12})[Q(0,0,2)+Q(2,2,0)] + \frac{1}{4}r_{13}[Q(0,2,0)Q(2,0,2)] + \frac{1}{4}r_{23}[Q(0,2,2)+Q(2,0,0)] \tag{S7}$$

After use of symmetry to keep only non-equivalent Qs, this leads to

$$Q(0,0,1) = (1-r_{12})r_{23}Q(0,0,0) + \frac{1}{2}(1-r_{12})Q(0,0,2) + \frac{1}{2}r_{13}Q(0,2,0) + \frac{1}{2}r_{23}Q(0,2,2)$$

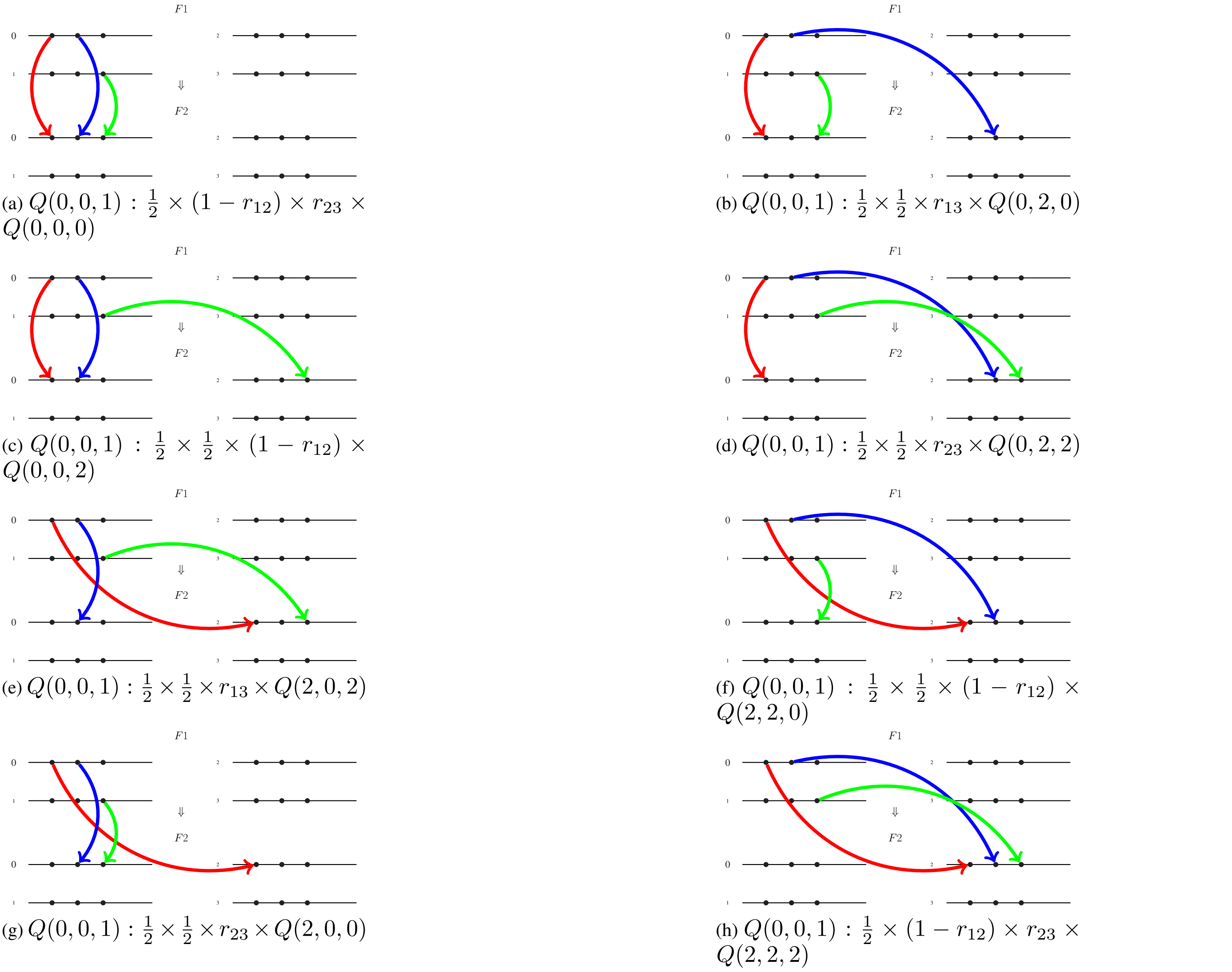

(a) $Q(0,0,1) : \frac{1}{2} \times (1-r_{12}) \times r_{23} \times Q(0,0,0)$

(b) $Q(0,0,1) : \frac{1}{2} \times \frac{1}{2} \times r_{13} \times Q(0,2,0)$

(c) $Q(0,0,1) : \frac{1}{2} \times \frac{1}{2} \times (1-r_{12}) \times Q(0,0,2)$

(d) $Q(0,0,1) : \frac{1}{2} \times \frac{1}{2} \times r_{23} \times Q(0,2,2)$

(e) $Q(0,0,1) : \frac{1}{2} \times \frac{1}{2} \times r_{13} \times Q(2,0,2)$

(f) $Q(0,0,1) : \frac{1}{2} \times \frac{1}{2} \times (1-r_{12}) \times Q(2,2,0)$

(g) $Q(0,0,1) : \frac{1}{2} \times \frac{1}{2} \times r_{23} \times Q(2,0,0)$

(h) $Q(0,0,1) : \frac{1}{2} \times (1-r_{12}) \times r_{23} \times Q(2,2,2)$

Figure S3: The graphical representation of the factors multiplying each Q on the right-hand side of Eq. S7 for $Q(0,0,1)$.

3.3 The self consistent equation for $Q(0, 0, 2)$

Figure S4 displays the 8 factors in the self-consistent equation for $Q(0, 0, 2)$:

$$Q(0,0,2) = \tfrac{1}{4}(1 - r_{12})[Q(0,0,1) + Q(2,2,3)] + \tfrac{1}{4}(1 - r_{12})[Q(0,0,3) + Q(2,2,1)] + \tfrac{1}{8}[Q(0,2,1) + Q(2,0,3)] + \tfrac{1}{8}[Q(0,2,3) + Q(2,0,1)] \tag{S8}$$

After use of symmetry to keep only non-equivalent Qs, this leads to

$$Q(0,0,2) = \tfrac{1}{2}(1 - r_{12})Q(0,0,1) + \tfrac{1}{2}(1 - r_{12})Q(0,0,2) + \tfrac{1}{4}Q(0,2,1) + \tfrac{1}{4}Q(0,2,3)$$

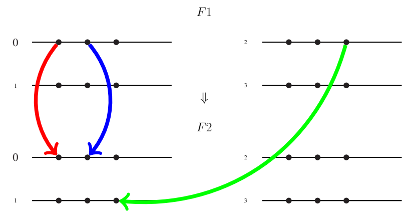

(a) $Q(0,0,2)\ :\ \frac{1}{2} \times (1 - r_{12}) \times \frac{1}{2} \times Q(0,0,1)$

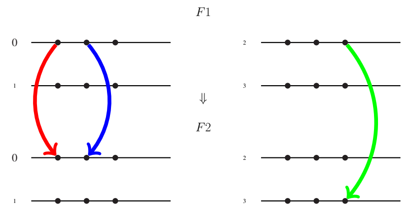

(b) $Q(0,0,2)\ :\ \frac{1}{2} \times (1 - r_{12}) \times \frac{1}{2} \times Q(0,0,3)$

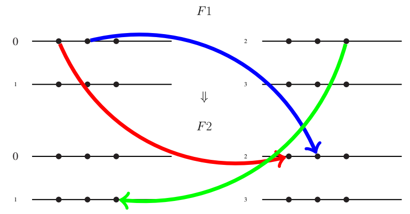

(c) $Q(0,0,2)\ :\ \frac{1}{2} \times (1 - r_{12}) \times \frac{1}{2} \times Q(2,2,1)$

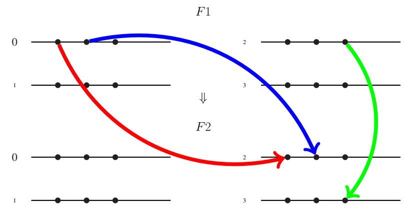

(d) $Q(0,0,2)\ :\ \frac{1}{2} \times (1 - r_{12}) \times \frac{1}{2} \times Q(2,2,3)$

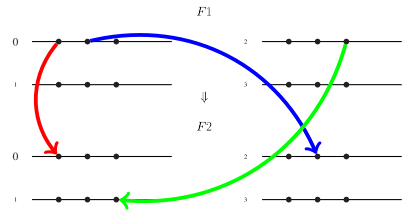

(e) $Q(0,0,2) : \frac{1}{2} \times \frac{1}{2} \times \frac{1}{2} \times Q(0,2,1)$

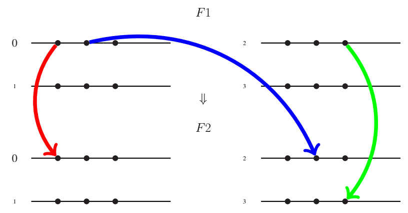

(f) $Q(0,0,2) : \frac{1}{2} \times \frac{1}{2} \times \frac{1}{2} \times Q(0,2,3)$

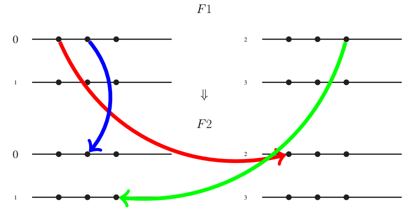

(g) $Q(0,0,2) : \frac{1}{2} \times \frac{1}{2} \times \frac{1}{2} \times Q(2,0,1)$

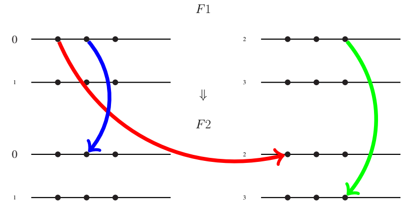

(h) $Q(0,0,2) : \frac{1}{2} \times \frac{1}{2} \times \frac{1}{2} \times Q(2,0,3)$

Figure S4: The graphical representation of the factors multiplying each Q on the right-hand side of Eq. S8 for $Q(0, 0, 2)$.

3.4 The self consistent equation for $Q(0,1,0)$

Figure S5 displays the 8 factors in the self-consistent equation for $Q(0,1,0)$:

$$Q(0,1,0) = \tfrac{1}{2}r_{12}r_{23}[Q(0,0,0)+Q(2,2,2)] + \tfrac{1}{4}r_{12}[Q(0,0,2)+Q(2,2,0)] + \tfrac{1}{4}(1-r_{13})[Q(0,2,0)+Q(2,0,2)] + \tfrac{1}{4}r_{23}[Q(0,2,2)+Q(2,0,0)] \qquad \text{(S9)}$$

After use of symmetry to keep only non-equivalent Qs, this leads to

$$Q(0,1,0) = r_{12}r_{23}Q(0,0,0) + \tfrac{1}{2}r_{12}Q(0,0,2) + \tfrac{1}{2}(1-r_{13})Q(0,2,0) + \tfrac{1}{2}r_{23}Q(0,2,2)$$

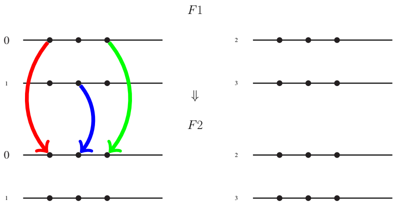

(a) $Q(0,1,0) : \frac{1}{2} \times r_{12} \times (1-r_{23}) \times Q(0,0,0)$

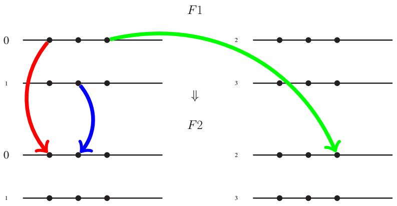

(b) $Q(0,1,0) : \frac{1}{2} \times r_{12} \times \frac{1}{2} \times Q(0,0,2)$

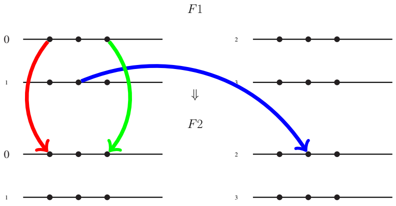

(c) $Q(0,1,0) : \frac{1}{2} \times \frac{1}{2} \times r_{13} \times Q(0,2,0)$

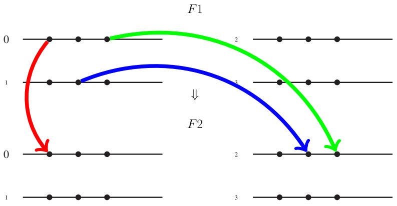

(d) $Q(0,1,0) : \frac{1}{2} \times \frac{1}{2} \times r_{23} \times Q(0,2,2)$

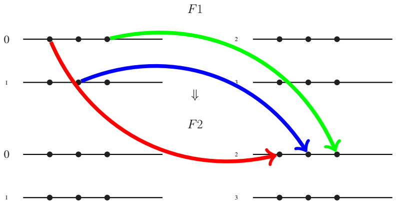

(e) $Q(0,1,0) : \frac{1}{2} \times r_{12}r_{23} \times Q(2,2,2)$

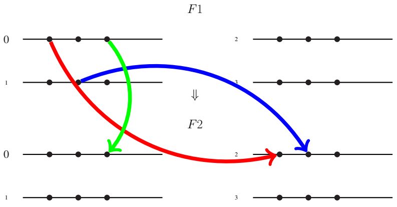

(f) $Q(0,1,0) : \frac{1}{2} \times \frac{1}{2} \times r_{12} \times Q(2,2,0)$

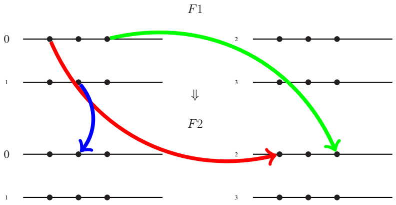

(g) $Q(0,1,0) : \frac{1}{2} \times \frac{1}{2} \times (1-r_{13}) \times Q(2,0,2)$

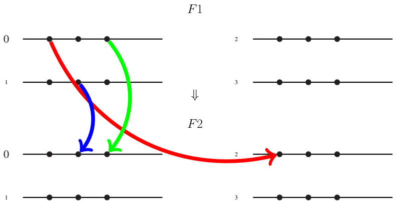

(h) $Q(0,1,0) : \frac{1}{2} \times r_{23} \times \frac{1}{2} \times Q(2,0,0)$

Figure S5: The graphical representation of the factors multiplying each Q on the right-hand side of Eq. S9 for $Q(0,1,0)$.

3.5 The self consistent equation for $Q(0,1,1)$

Figure S6 displays the 8 factors in the self-consistent equation for $Q(0,1,1)$:

$$Q(0,1,1) = \frac{1}{2}r_{12}(1-r_{23})[Q(0,0,0)+Q(2,2,2)] + \frac{1}{4}r_{12}[Q(0,0,2)+Q(2,2,0)] + \frac{1}{4}r_{13}[Q(0,2,0)+Q(2,0,2)] + \frac{1}{4}(1-r_{23})[Q(0,2,2)+Q(2,0,0)] \quad \text{(S10)}$$

After use of symmetry to keep only non-equivalent Qs, this leads to

$$Q(0,1,1) = r_{12}(1-r_{23})Q(0,0,0) + \frac{1}{2}r_{12}Q(0,0,2) + \frac{1}{2}r_{13}Q(0,2,0) + \frac{1}{2}(1-r_{23})Q(0,2,2)$$

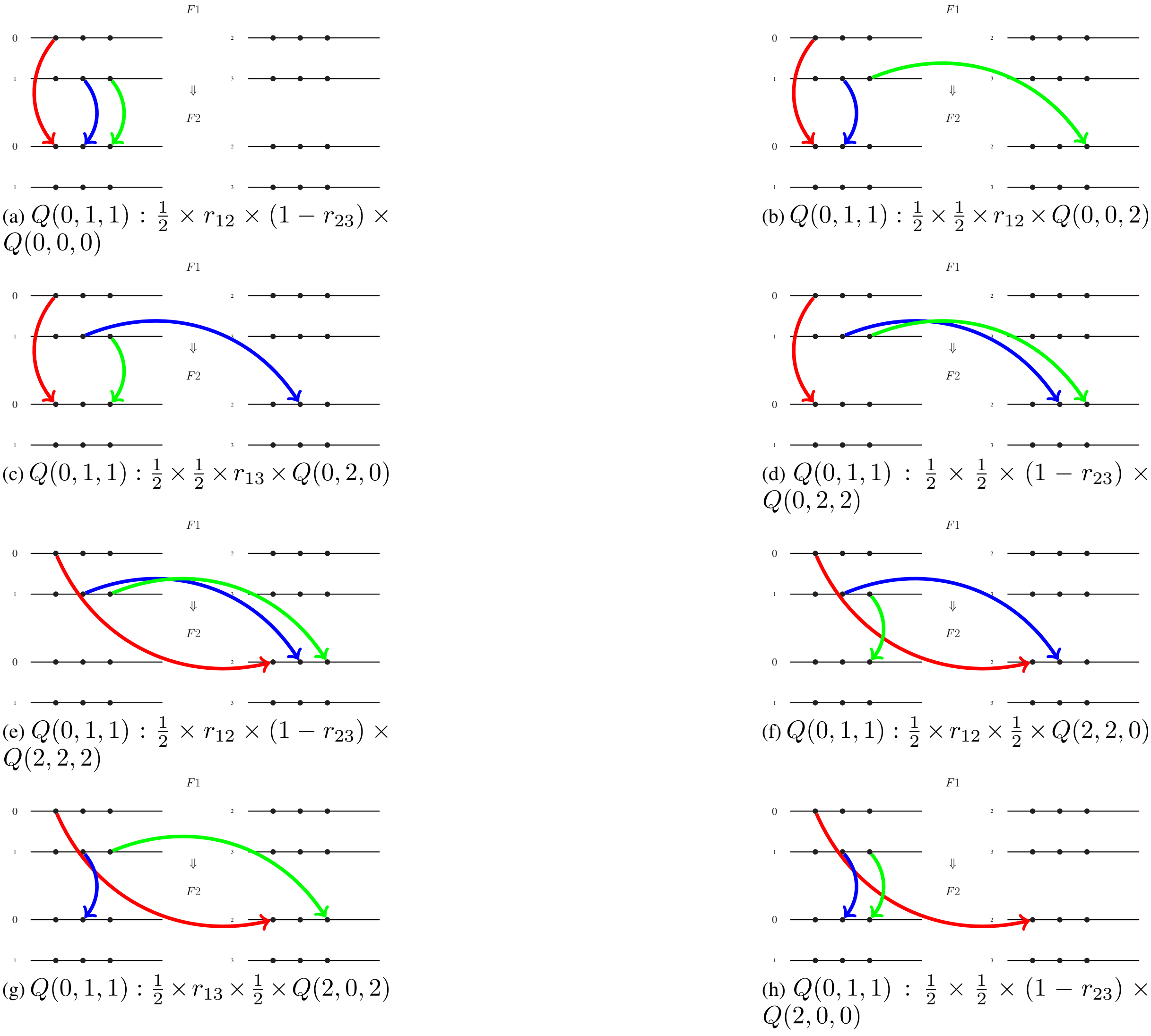

(a) $Q(0,1,1) : \frac{1}{2} \times r_{12} \times (1-r_{23}) \times Q(0,0,0)$

(b) $Q(0,1,1) : \frac{1}{2} \times \frac{1}{2} \times r_{12} \times Q(0,0,2)$

(c) $Q(0,1,1) : \frac{1}{2} \times \frac{1}{2} \times r_{13} \times Q(0,2,0)$

(d) $Q(0,1,1) : \frac{1}{2} \times \frac{1}{2} \times (1-r_{23}) \times Q(0,2,2)$

(e) $Q(0,1,1) : \frac{1}{2} \times r_{12} \times (1-r_{23}) \times Q(2,2,2)$

(f) $Q(0,1,1) : \frac{1}{2} \times r_{12} \times \frac{1}{2} \times Q(2,2,0)$

(g) $Q(0,1,1) : \frac{1}{2} \times r_{13} \times \frac{1}{2} \times Q(2,0,2)$

(h) $Q(0,1,1) : \frac{1}{2} \times \frac{1}{2} \times (1-r_{23}) \times Q(2,0,0)$

Figure S6: The graphical representation of the factors multiplying each Q on the right-hand side of Eq. S10 for $Q(0,1,1)$.

3.6 The self consistent equation for $Q(0, 1, 2)$

Figure S7 displays the 8 factors in the self-consistent equation for $Q(0, 1, 2)$:

$$Q(0,1,2) = \tfrac{1}{4}r_{12}[Q(0,0,1)+Q(2,2,3)] + \tfrac{1}{4}r_{12}[Q(0,0,3)+Q(2,2,1)] + \tfrac{1}{8}[Q(0,2,1)+Q(2,0,3)] + \tfrac{1}{8}[Q(0,2,3)+Q(2,0,1)] \qquad \text{(S11)}$$

After use of symmetry to keep only non-equivalent Qs, this leads to

$$Q(0,1,2) = \tfrac{1}{2}r_{12}Q(0,0,1) + \tfrac{1}{2}r_{12}Q(0,0,2) + \tfrac{1}{4}Q(0,2,1) + \tfrac{1}{4}Q(0,2,3)$$

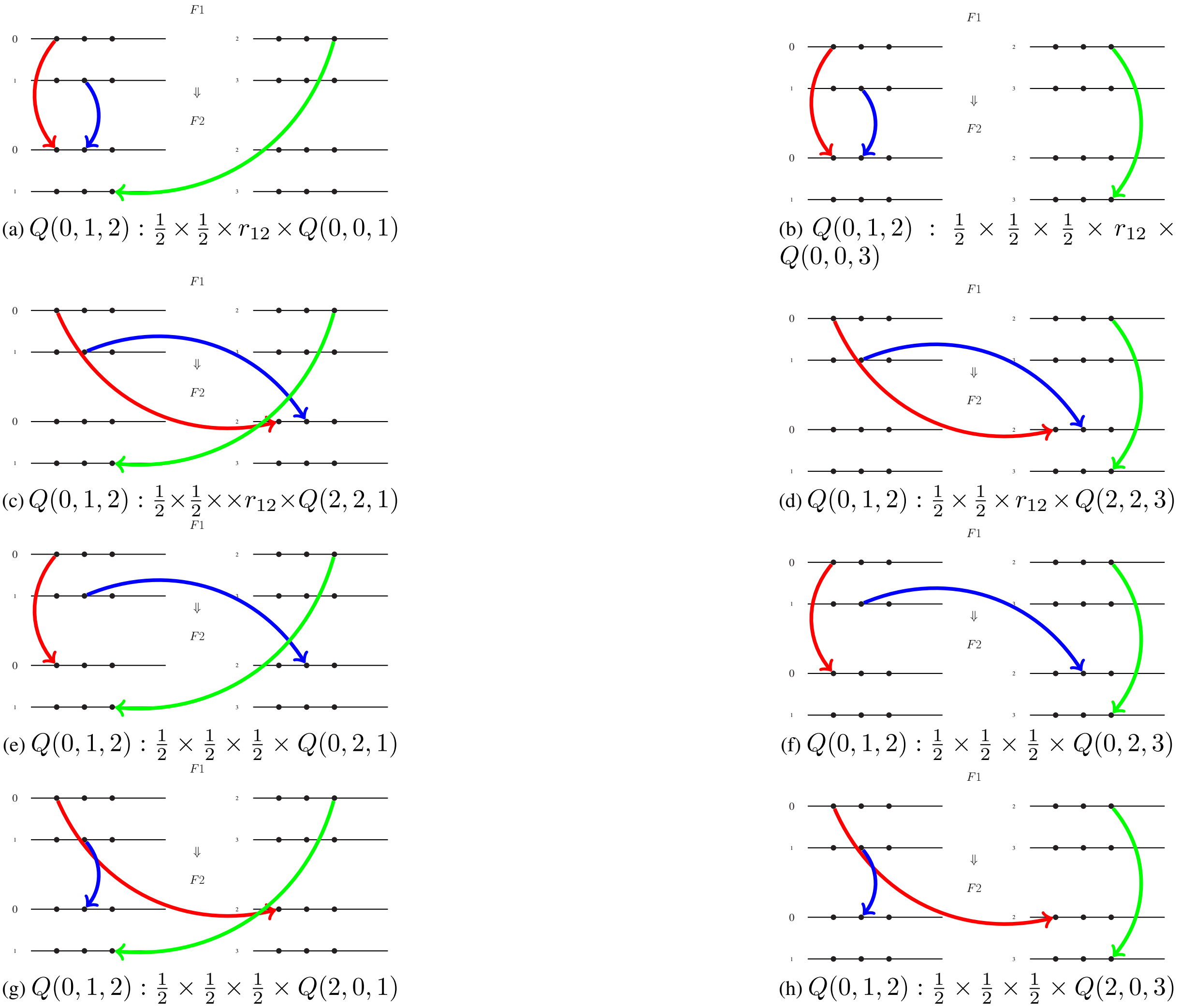

(a) $Q(0,1,2) : \frac{1}{2} \times \frac{1}{2} \times r_{12} \times Q(0,0,1)$

(b) $Q(0,1,2) : \frac{1}{2} \times \frac{1}{2} \times \frac{1}{2} \times r_{12} \times Q(0,0,3)$

(c) $Q(0,1,2) : \frac{1}{2} \times \frac{1}{2} \times \times r_{12} \times Q(2,2,1)$

(d) $Q(0,1,2) : \frac{1}{2} \times \frac{1}{2} \times r_{12} \times Q(2,2,3)$

(e) $Q(0,1,2) : \frac{1}{2} \times \frac{1}{2} \times \frac{1}{2} \times Q(0,2,1)$

(f) $Q(0,1,2) : \frac{1}{2} \times \frac{1}{2} \times \frac{1}{2} \times Q(0,2,3)$

(g) $Q(0,1,2) : \frac{1}{2} \times \frac{1}{2} \times \frac{1}{2} \times Q(2,0,1)$

(h) $Q(0,1,2) : \frac{1}{2} \times \frac{1}{2} \times \frac{1}{2} \times Q(2,0,3)$

Figure S7: The graphical representation of the factors multiplying each Q on the right-hand side of Eq. S11 for $Q(0, 1, 2)$.

3.7 The self consistent equation for $Q(0, 2, 0)$

Figure S8 displays the 8 factors in the self-consistent equation for $Q(0, 2, 0)$:

$$Q(0,2,0) = \tfrac{1}{4}(1 - r_{13})[Q(0,1,0) + Q(2,3,2)] + \tfrac{1}{8}[Q(0,1,2) + Q(2,3,0)] + \tfrac{1}{4}(1 - r_{13})[Q(0,3,0) + Q(2,1,2)] + \tfrac{1}{8}[Q(0,1,3) + Q(2,1,0)] \tag{S12}$$

After use of symmetry to keep only non-equivalent Qs, this leads to

$$Q(0,2,0) = \tfrac{1}{2}(1 - r_{13})Q(0,1,0) + \tfrac{1}{4}Q(0,1,2) + \tfrac{1}{2}(1 - r_{13})Q(0,2,0) + \tfrac{1}{4}Q(0,1,2)$$

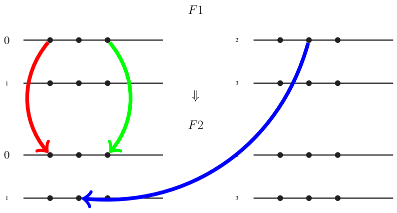

(a) $Q(0,2,0)\ :\ \frac{1}{2} \times (1 - r_{13}) \times \frac{1}{2} \times Q(0,1,0)$

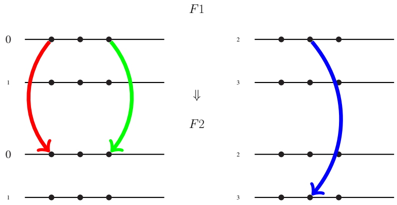

(b) $Q(0,2,0)\ :\ \frac{1}{2} \times (1 - r_{13}) \times \frac{1}{2} \times Q(0,3,0)$

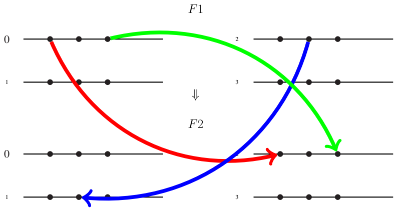

(c) $Q(0,2,0)\ :\ \frac{1}{2} \times (1 - r_{13}) \times \frac{1}{2} \times Q(2,1,2)$

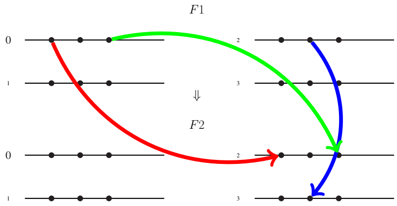

(d) $Q(0,2,0)\ :\ \frac{1}{2} \times (1 - r_{13}) \times \frac{1}{2} \times Q(2,3,2)$

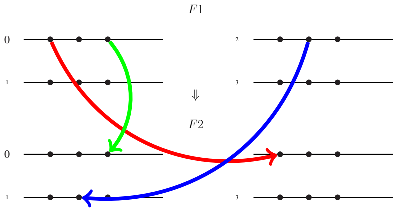

(e) $Q(0,2,0) : \frac{1}{2} \times \frac{1}{2} \times \frac{1}{2} \times Q(2,1,0)$

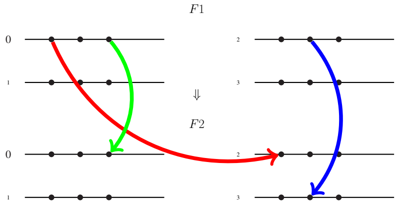

(f) $Q(0,2,0) : \frac{1}{2} \times \frac{1}{2} \times \frac{1}{2} \times Q(2,3,0)$

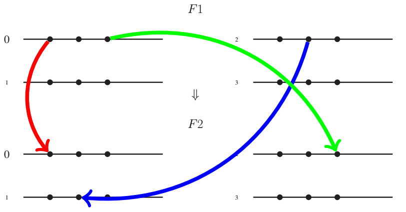

(g) $Q(0,2,0) : \frac{1}{2} \times \frac{1}{2} \times \frac{1}{2} \times Q(0,1,2)$

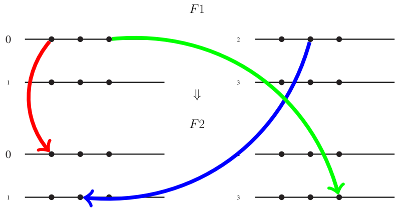

(h) $Q(0,2,0) : \frac{1}{2} \times \frac{1}{2} \times \frac{1}{2} \times Q(0,1,3)$

Figure S8: The graphical representation of the factors multiplying each Q on the right-hand side of Eq. S12 for $Q(0,2,0)$.

3.8 The self consistent equation for $Q(0, 2, 1)$

Figure S9 displays the 8 factors in the self-consistent equation for $Q(0, 2, 1)$:

$$Q(0,2,1) = \tfrac{1}{4}r_{13}[Q(0,1,0) + Q(2,3,2)] + \tfrac{1}{8}[Q(0,1,2) + Q(2,3,0)] + \tfrac{1}{4}r_{13}[Q(0,3,0) + Q(2,1,2)] + \tfrac{1}{8}[Q(0,3,2) + Q(2,1,0)] \quad \text{(S13)}$$

After use of symmetry to keep only non-equivalent Qs, this leads to

$$Q(0,2,1) = \tfrac{1}{2}r_{13}Q(0,1,0) + \tfrac{1}{4}Q(0,1,2) + \tfrac{1}{2}r_{13}Q(0,2,0) + \tfrac{1}{4}Q(0,2,3)$$

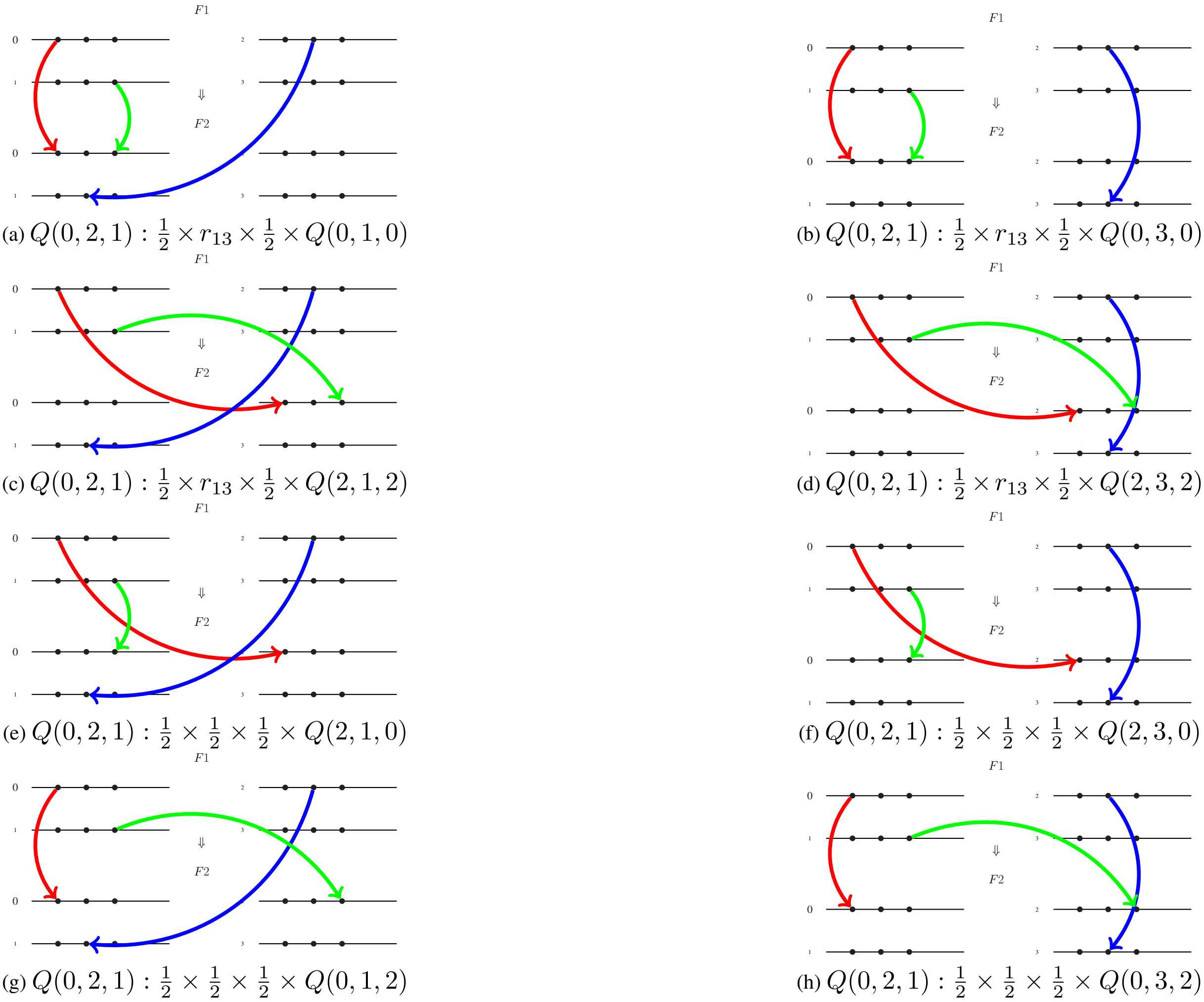

(a) $Q(0,2,1) : \frac{1}{2} \times r_{13} \times \frac{1}{2} \times Q(0,1,0)$

(b) $Q(0,2,1) : \frac{1}{2} \times r_{13} \times \frac{1}{2} \times Q(0,3,0)$

(c) $Q(0,2,1) : \frac{1}{2} \times r_{13} \times \frac{1}{2} \times Q(2,1,2)$

(d) $Q(0,2,1) : \frac{1}{2} \times r_{13} \times \frac{1}{2} \times Q(2,3,2)$

(e) $Q(0,2,1) : \frac{1}{2} \times \frac{1}{2} \times \frac{1}{2} \times Q(2,1,0)$

(f) $Q(0,2,1) : \frac{1}{2} \times \frac{1}{2} \times \frac{1}{2} \times Q(2,3,0)$

(g) $Q(0,2,1) : \frac{1}{2} \times \frac{1}{2} \times \frac{1}{2} \times Q(0,1,2)$

(h) $Q(0,2,1) : \frac{1}{2} \times \frac{1}{2} \times \frac{1}{2} \times Q(0,3,2)$

Figure S9: The graphical representation of the factors multiplying each Q on the right-hand side of Eq. S13 for $Q(0, 2, 1)$.

3.9 The self consistent equation for $Q(0, 2, 2)$

Figure S10 displays the 8 factors in the self-consistent equation for $Q(0, 2, 2)$:

$$Q(0,2,2) = \tfrac{1}{4}(1-r_{23})[Q(0,1,1)+Q(2,3,3)] + \tfrac{1}{8}[Q(0,1,3)+Q(2,3,1)] + \tfrac{1}{8}[Q(0,3,1)+Q(2,1,3)] + \tfrac{1}{4}(1-r_{23})[Q(0,3,3)+Q(2,1,1)] \tag{S14}$$

After use of symmetry to keep only non-equivalent Qs, this leads to

$$Q(0,2,2) = \tfrac{1}{2}(1-r_{23})Q(0,1,1) + \tfrac{1}{4}Q(0,1,2) + \tfrac{1}{4}Q(0,2,1) + \tfrac{1}{2}(1-r_{23})Q(0,2,2)$$

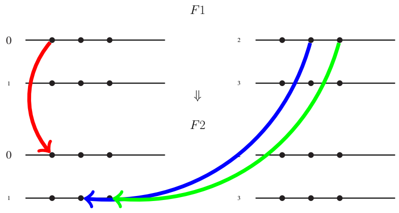

(a) $Q(0,2,2)\ :\ \frac{1}{2} \times \frac{1}{2} \times (1-r_{23}) \times Q(0,1,1)$

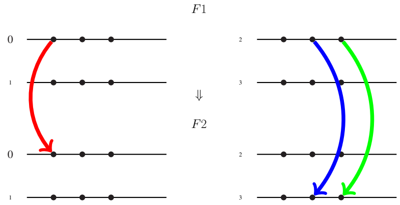

(b) $Q(0,2,2)\ :\ \frac{1}{2} \times \frac{1}{2} \times (1-r_{23}) \times Q(0,3,3)$

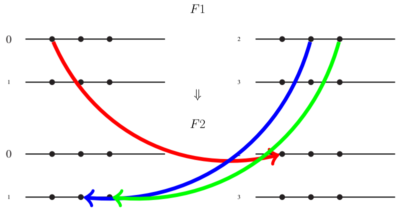

(c) $Q(0,2,2)\ :\ \frac{1}{2} \times \frac{1}{2} \times (1-r_{23}) \times Q(2,1,1)$

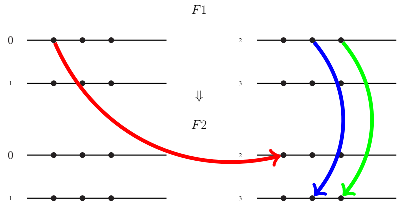

(d) $Q(0,2,2)\ :\ \frac{1}{2} \times \frac{1}{2} \times (1-r_{23}) \times Q(2,3,3)$

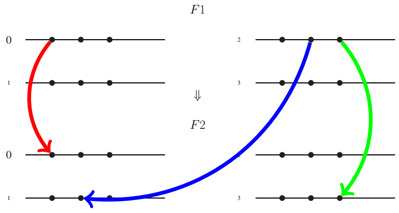

(e) $Q(0,2,2) : \frac{1}{2} \times \frac{1}{2} \times \frac{1}{2} \times Q(0,1,3)$

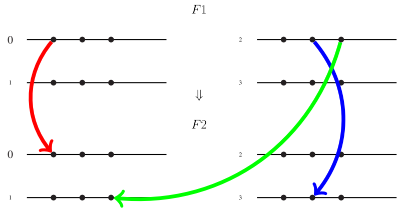

(f) $Q(0,2,2) : \frac{1}{2} \times \frac{1}{2} \times \frac{1}{2} \times Q(0,3,1)$

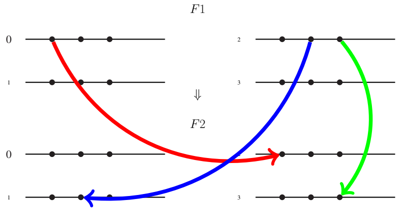

(g) $Q(0,2,2) : \frac{1}{2} \times \frac{1}{2} \times \frac{1}{2} \times Q(2,1,3)$

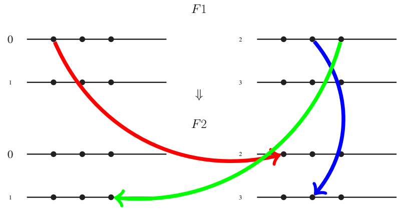

(h) $Q(0,2,2) : \frac{1}{2} \times \frac{1}{2} \times \frac{1}{2} \times Q(2,3,1)$

Figure S10: The graphical representation of the factors multiplying each Q on the right-hand side of Eq. S14 for $Q(0, 2, 2)$.

3.10 The self consistent equation for $Q(0, 2, 3)$

Figure S11 displays the 8 factors in the self-consistent equation for $Q(0, 2, 3)$:

$$Q(0,2,3) = \frac{1}{4}r_{23}[Q(0,1,1) + Q(2,3,3)] + \frac{1}{8}[Q(0,1,3) + Q(2,3,1)] + \frac{1}{8}[Q(0,2,1) + Q(2,1,3)] + \frac{1}{4}r_{23}[Q(0,3,3) + Q(2,1,1)] \quad \text{(S15)}$$

After use of symmetry to keep only non-equivalent Qs, this leads to

$$Q(0,2,3) = \frac{1}{2}r_{23}Q(0,1,1) + \frac{1}{4}Q(0,1,2) + \frac{1}{4}Q(0,2,1) + \frac{1}{2}r_{23}Q(0,2,2)$$

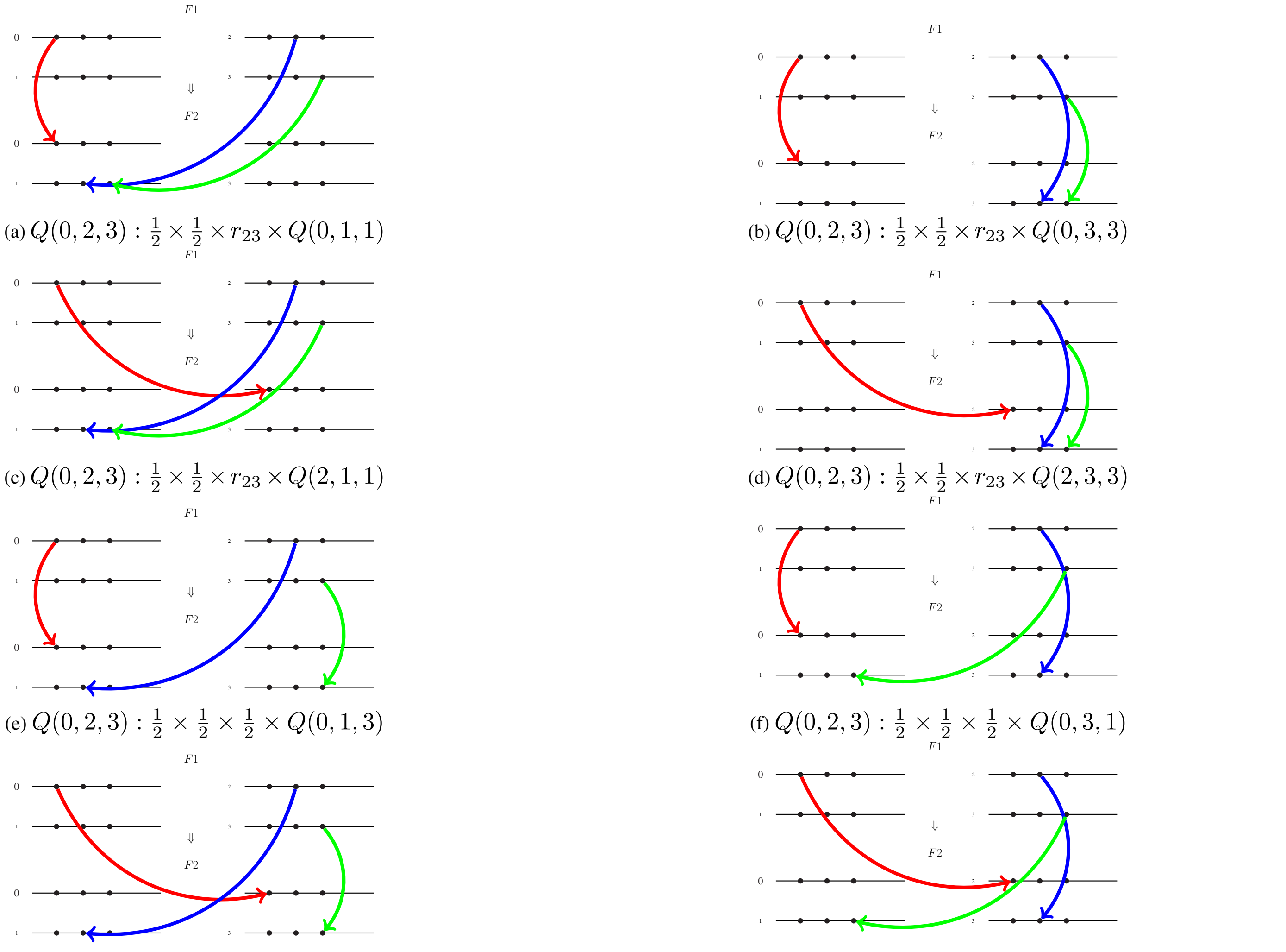

(a) $Q(0,2,3) : \frac{1}{2} \times \frac{1}{2} \times r_{23} \times Q(0,1,1)$

(b) $Q(0,2,3) : \frac{1}{2} \times \frac{1}{2} \times r_{23} \times Q(0,3,3)$

(c) $Q(0,2,3) : \frac{1}{2} \times \frac{1}{2} \times r_{23} \times Q(2,1,1)$

(d) $Q(0,2,3) : \frac{1}{2} \times \frac{1}{2} \times r_{23} \times Q(2,3,3)$

(e) $Q(0,2,3) : \frac{1}{2} \times \frac{1}{2} \times \frac{1}{2} \times Q(0,1,3)$

(f) $Q(0,2,3) : \frac{1}{2} \times \frac{1}{2} \times \frac{1}{2} \times Q(0,3,1)$

(g) $Q(0,2,3) : \frac{1}{2} \times \frac{1}{2} \times \frac{1}{2} \times Q(2,1,3)$

(h) $Q(0,2,3) : \frac{1}{2} \times \frac{1}{2} \times \frac{1}{2} \times Q(2,3,1)$

Figure S11: The graphical representation of the factors multiplying each Q on the right-hand side of Eq. S15 for $Q(0, 2, 3)$.
